# Induced Pluripotent Stem Cells to Understand Mucopolysaccharidosis. I: Demonstration of a Migration Defect in Neural Precursors

**DOI:** 10.3390/cells9122593

**Published:** 2020-12-03

**Authors:** Silvin Lito, Adama Sidibe, Sten Ilmjarv, Patricie Burda, Matthias Baumgartner, Bernhard Wehrle-Haller, Karl-Heinz Krause, Antoine Marteyn

**Affiliations:** 1 Department of Pathology and Immunology, Faculty of Medicine, University of Geneva, CH-1211 Geneva, Switzerland; silvin.Lito@unige.ch (S.L.); sten.Ilmjarv@unige.ch (S.I.); antoine.marteyn@unige.ch (A.M.); 2 Department of Cell Physiology and Metabolism, Faculty of Medicine, University of Geneva, CH-1211 Geneva, Switzerland; adama.sidibe@unige.ch (A.S.); bernhard.wehrle-haller@unige.ch (B.W.-H.); 3 Division of Metabolism and Children’s Research Center, University Children’s Hospital, CH-8032 Zürich, Switzerland; patricie.burda@kispi.uzh.ch (P.B.); matthias.baumgartner@kispi.uzh.ch (M.B.)

**Keywords:** induced pluripotent stem cells, neuronal differentiation, disease modelling, mucopolysaccharidosis I, neural migration, neurite outgrowth

## Abstract

*Background*: Mucopolysaccharidosis type I-Hurler (MPS1-H) is a severe genetic lysosomal storage disorder due to loss-of-function mutations in the *IDUA* gene. The subsequent complete deficiency of alpha l-iduronidase enzyme is directly responsible of a progressive accumulation of glycosaminoglycans (GAG) in lysosomes which affects the functions of many tissues. Consequently, MPS1 is characterized by systemic symptoms (multiorgan dysfunction) including respiratory and cardiac dysfunctions, skeletal abnormalities and early fatal neurodegeneration. *Methods*: To understand mechanisms underlying MPS1 neuropathology, we generated induced pluripotent stem cells (iPSC) from a MPS1-H patient with loss-of-function mutations in both *IDUA* alleles. To avoid variability due to different genetic background of iPSC, we established an isogenic control iPSC line by rescuing IDUA expression by a lentivectoral approach. *Results*: Marked differences between MPS1-H and *IDUA*-corrected isogenic controls were observed upon neural differentiation. A scratch assay revealed a strong migration defect of MPS1-H cells. Also, there was a massive impact of *IDUA* deficiency on gene expression (340 genes with an FDR < 0.05). *Conclusions*: Our results demonstrate a hitherto unknown connection between lysosomal degradation, gene expression and neural motility, which might account at least in part for the phenotype of MPS1-H patients.

## 1. Introduction

Mucopolysaccharidosis type I-Hurler (MPS1-H) is a rare inherited metabolic disorder caused by loss-of-function mutations in the *IDUA* gene leading to the complete deficiency of alpha-L-iduronidase, the enzyme involved in the degradation of glycosaminoglycans (GAGs) [1,2]. The progressive intralysosomal accumulation of dermatan sulfate (DS) and heparane sulfate (HS) in tissues is directly linked to multiorgan dysfunction [2]. So, the mucopolysaccharidosis type I-Hurler (MPS1-H), which is the most prevalent phenotype and the most severe form of MPS1, is characterized by critical musculoskeletal alterations, cardiovascular, respiratory affections and serious neurological dysfunctions [3,4,5,6]. Despite this broad phenotypic spectrum [2], the progression of MPS1-H pathological process is insufficiently advanced at birth to detect any phenotypic alteration. Indeed, children with Hurler’s syndrome (MPS1-H) do not develop specific clinical features such as coarse facies traits or hernias until 3–6 months of age [7,8]. Likewise, cognitive abnormalities are rarely detected during the first year of life [2]. This delay of pathological manifestations is problematic as the progression of MPS1-H is rapid and death usually occurs prematurely within the first decade of age if left untreated [9,10]. The success of therapeutic treatments, which is essentially based on hematopoietic stem cells transplantation (HSCT) [11] is often correlated to an early diagnostic [12]. If MPS1-H patients are treated before the progressive decline, usually starting around 2 years-old [13], their lifespan can be prolonged, irreversible neurological damages can be limited and consequently their intelligence quotient (IQ) score stabilized [6,14,15].

Although these neurological defects are also detected in MPS2, MPS3 and in some case of other subtype of MPS1, they are more varied and severe in MPS1-H [16]. Thus, MPS1-H patients can present sleeping disorders, behavioral problems, limited language, hearing loss [2] and cognitive impairment [2,3,5,6,16]. Although some of these disorders are directly derived from developmental abnormalities such as the limited language which may resulted from both hearing loss and enlargement of the tongue [2,17], the causes of other neurological symptoms remain poorly understood.

Some studies, carried out using fibroblasts [18] or iPSCs [19], isolated or derived from MPS3 patients, have highlighted alterations of cell migration and neuritogenesis. These defects, also observed in MPS3 mice [20], would result from HS proteoglycan accumulation which would disrupt normal brain development. Moreover, the use and the characterization of different mouse models of MPS1 and MPS3 revealed a secondary accumulation of gangliosides (GM2 and GM3) in the central nervous system (CNS) of MPS mice [21,22,23]. Since this ganglioside storage has also been observed in the brains of patients affected by different forms of MPS [24,25], other studies explored the probable effects of excessive gangliosides in the pathological process of MPS1 [26] and hypothesized its possible involvement in the onset of hyperactive behavior [27,28], and sleeping disorders due to alteration of circadian rhythms [29]. Nevertheless, other unknown neuropathological mechanisms must be implicated [23].

In this study, we took advantage of the potential of induced pluripotent stem cells (iPSC), derived from a MPS1-H child [30], to reproduce, in vitro, the different steps observed during neural development and to identify any cellular and molecular defects responsible of neuropathological manifestation. To counteract the major cause of transcriptional variation resulting from the genetic background difference between individual lines [31], we generated an isogenic control iPSC line, constitutively expressing the alpha-l-iduronidase. Both MPS1-H and rescue MPS1-H (rMPS1-H) iPSCs were differentiated into neuronal lineage. Analysis of RNA sequencing (RNA-seq), which was performed on 3-weeks old neurospheres derived from these iPSCs lines, allowed the identification of clusters of genes pointing towards migration defects and neuroanatomical defects. The alteration of migration was validated, in vitro, by the performance of a scratch test on neural stem/progenitor cells (NSCs) derived from MPS1-H iPSCs. In addition, maturation of these MPS1-H NSCs highlighted a defective neurite outgrowth. All molecular and cellular alterations identified in MPS1-H neural cells may explain some of neuropathological features described in MPS1-H patients.

## 2. Materials and Methods

### 2.1. Construction of Plasmid and Lentiviral Vector

*IDUA* cDNA ORF expression clone NM_000203.4 (GeneCopoeia, Rockville, MD, USA) was cloned in GATEWAY™ Entry plasmid by PCR (Herculase II Fusion DNA polymerase, Agilent, Santa Clara, CA, USA) by using Fwd: 5′-GGGGACAAGTTTGTACAAAAAAGCAGGCTTCACC ATGCGTCCCCTGCGCCCCCG-3′ and Rev: 5′-GGGGACCACTTTGTACAAGAAAGCTGGGT TCATGGATTGCCCGGGGATGGGGG-3′ primers. The final lentivector plasmid was assembled by a GATEWAY™ LR Clonase™ II (ThermoFisher, Reinach, Switzerland), mediated recombination of a pENTR plasmid containing the human UBI and a lentivector destination cassette containing an additional transcription unit encoding for blasticidin resistance gene upon human PGK promoter. Final lentivector was produced by transient transfection of HEK 293T cells with the generated lentivector plasmid pCWX-UBI-IDUA-PGK-BSD, the pCAG-VSVG envelope plasmid and the psPAX2 plasmid encoding gag/pol, following the CaPO_4_ method [32].

### 2.2. Lentiviral Vector Transduction and Rescue

α-l-Iduronidase enzyme (IDUAα) activity have been both restored in MPS1-H fibroblasts, MPS1-H iPSCs and MPS1-H iPSCs-derived NSCs by using UBI-IDUA-PGK-BSD lentivector. One day before the transduction, MPS1-H cells were seeded at 33% of confluency on 6 well plates. Cells were transduced using a MOI of 5. Cells were cultured for 5 days before starting blasticidin selection. 5 µg/mL of blasticidin was added to the medium during 7 days. Culture medium was changed daily.

### 2.3. IDUA Activity Measurement Assay

The IDUAα activity assay was performed by fluorimetric determination using 4MU α-L-iduronide on cell lysate by [33] modified protocol. One µg of protein interacted with 7 µg of 4MU α L-iduronide (Glycosynth, Cheshire, UK), 2.5 µg of saccharonolactone (Sigma-Aldrich, Buchs, Switzerland) at 37 °C, 5% CO_2_ during 20 min on a shaking platform. Reaction stop is given by pH10 glycine buffer (Sigma-Aldrich, Buchs, Switzerland). Measurement was made by PARADIGM reader (Molecular Devices, Sunnyvale, CA, USA) at λ_ex_ = 355 nm, λ_em_ = 465 nm. IDUAα was calculated using fluorescence extrapolating from 50 nM 4MU standard solution (Sigma-Aldrich, Buchs, Switzerland).

### 2.4. Establishment and Characterization of MPS1-H iPSC Line

The MPS1-H iPSC line was generated from the reprogramming of dermal fibroblasts, isolated from a skin biopsy of 2-year-old male MPS1-H child [30]. The loss-of-function of IDUAα have been associated to the presence of two distinct mutations distributed on the two alleles of the *IDUA* gene: NM_000203.5(IDUA):c.1073_1093del (p.His358_Thr364del) and NM_000203.4(IDUA):c.1205 G>A (p.Trp402Ter). All details concerning reprogramming, cell culture maintenance and characterization of the line have been reported in a previous study [30]. The isogenic control iPSC line, resulting from the transduction of MPS1-H iPSC line with a lentivector allowing the expression of normal *IDUA* gene, have been characterized in similar experimental conditions. The tree clones S1, S2 and S4 of MPS1-H iPSCs were rescued. The clone S3 was not rescued since its genome integrity had not been validated by a previous comparative genomic hybridization (CGH) array.

### 2.5. hiPSC-Derived NSCs

hiPS clones were differentiated in vitro toward neuronal lineage using a monolayer based differentiation protocol and DUAL SMAD inhibition [34]. Cells were cultured for 15 days, passaged on Matrigel-coated flasks at 5.5 × 10^5^ cells/cm^2^.

### 2.6. NSCs Culture

Cells (5.5 × 10^5^/cm^2^) were plated on pre-coated poly-L-ornithine/laminin dishes (2 µg/cm^2^) in Neurobasal medium supplemented with 2 mM L-glutamine, B27 supplement 0.2% final, NEAA (Life Technologies, Europe BV, Zug, Switzerland). 10 ng/mL of fibroblast growth factor 2 (FGF2; R&D systems) and epidermal growth factor (EGF; Life Technologies Europe BV, Zug, Switzerland) was added to the medium to promote proliferation. Medium change occurred every other day.

### 2.7. NSC Differentiation

Differentiation was initiated when NSCs reached 80% confluency. Both FGF2 and EGF were then removed from medium. Medium change was performed every 2–3 days. On day 10, cells were detached with accutase^®^ (Invitrogen, Basel, Switzerland) for 5 min at 37 °C, before to be plated on poly-L-ornithine/laminin coverslips at 158,000 cells/cm^2^. Cells were cultured in B27 neurobasal medium supplemented with 20 ng/mL of BDNF and GDNF (R&D Systems, Minneapolis, MN, USA) for additional 11 days. Only half of the medium was gently changed every 3–4 days.

### 2.8. Neurospheres Differentiation Protocol

MPS1-H iPSCs and rMPS1-H iPSCs were plated on Matrigel^®^ coated P150 flasks as described above. When iPSCs colonies reached 80% confluency, neural induction was promoted by switching the medium with X-vivo 10 medium (Lonza, Basel, Switzerland) supplemented with 0.5 µM of LDN193189 and 10 µM of SB431542. 10 µM of Y-27632 (Axon Medchem BV, Groningen, The Netherlands) was also added to the medium for 24 h. The day after (day 1), iPSCs were dissociated enzymatically with Accutase^®^ (Invitrogen) for 5 min at 37 °C and the resulting single-cell suspension was evenly distributed to 1000 cells/microwell of AggreWell™400 plates (Stemcell Technologies, Grenoble, France). At day 2, resulted uniform spheres were collected and were cultured in rotation (60 rpm, orbital shaker; Major Science, Saratoga, CA, USA), in uncoated six-well plates, to prevent spheres sticking together. Half of medium was then changed every 2 days following a strict schedule. At day 3, medium was substituted with B27 medium containing 0.5% BSA, and supplemented with LDN 0.5 µM, SB431542 10 µM. At day 8, SB431542 was removed from the medium while LDN was removed from day 9. From day 9, B27 neurobasal medium with 0.5% BSA was supplemented with 10 ng/mL of BDNF and GDNF, 200 µM of ascorbic acid and 1 µM of db-AMPc (Sigma-Aldrich, Buchs, Switzerland). At day 21, the neurospheres were analyzed.

### 2.9. Immunofluorescent Analysis

Coverslips were washed twice with HBSS+ CaCl_2_, MgCl_2_ (Gibco by Thermo Fisher Scientific, Waltham, MA, USA) and fixed with 3% paraformaldehyde (PFA) for 10 min at room temperature (RT). Permeabilization and antigen quenching was performed by incubating cells for 30 min at RT in blocking solution constituted by PBS containing 0.1% Triton X-100 and 3% BSA. Cells were then incubated overnight at 4 °C with primary antibodies: Mouse-anti-human MAP2 (Calbiochem by Sigma-Aldrich, Buchs, Switzerland) 1:500; Mouse-anti-human Ki-67 (Merck Millipore, Schaffhausen, Switzerland) 1:100; Mouse-anti-human LAMP-1 (H4A3, BD Pharmingen, San Diego, CA, USA) 1:500. Secondary antibodies were incubated for 1 h at RT. Hoechst staining was performed during incubation of secondary antibody at 5 µg/mL. Slides were mounted by FluorSave reagent (Calbiochem, EMD-Millipore, Schaffhausen, Switzerland). Images were captured with an LSM-800 Airyscan (Carl Zeiss AG, Felbach, Switzerland) microscope. Image acquisition was performed using ZEN software. Quantification and image processing were performed using MetaMorph and/or ImageJ. Neurite area and length was measured by ad hoc MathLab journal. Quality checks were performed manually, to ensure that artefacts did not interfere with measurements.

### 2.10. Scratch Test

Undifferentiated iPSCs-derived NSCs were seeded onto poly-L-ornithine/laminin pre-coated 96-well plate at a density of 6.25 × 10^5^ cells/cm^2^. The day after, the scratch was performed using a P200 pipette tip and NSCs medium was replaced. Plates were incubated at 37 °C, 5% CO_2_ and checked by ImageXpress widefield high-content analysis system every 12 h until the two migration fronts of the “scratch” met (48 h post-scratch). Scratch surface calculation was ensued by ImageJ software.

### 2.11. RNA Sequencing

RNA-seq was performed using triplicate of 3-week-old neurospheres derived from both MPS1-H iPSCs and rMPS1-H iPSCs. Total RNA was extracted according to the RNeasy Micro kit protocol (Qiagen, Hombrechtikon, Switzerland). RNA concentrations were measured by Qubit (Invitrogen) and their quality were validated by using the Bioanalyzer 2100 (Agilent) according to the manufacturer’s protocol. Stranded cDNA libraries resulted from mRNA by using Illumina TruSeq RNA library prep kit v2. 100 bp single-paired strands. High throughput sequencing was performed using HiSeq 4000 (Illumina) which resulted in 30 million copies per sample output on average (38–47 million). The sequencing quality control was done with FastQC v.0.11.5. The reads were mapped with the TopHat v.2.0.11 software to the UCSC hg38 human reference. Biological quality control and summarization were done with the PicardTools v.1.141. The table of counts with the number of reads mapping to each gene feature of UCSC mm10 was prepared with HTSeq v0.6p1 (htseq-count). Differential expression analysis was performed in R using the edgeR v. 3.14.0 package [35]. The *p*-value was corrected for multiple testing error using FDR (false discovery rate) with the Benjamini-Hochberg (BH) method. Genes with a *p*-value < 0.05 were considered differentially expressed.

## 3. Results

### 3.1. Restoration of IDUAα Activity of MPS1-H Cells Infected with UBI-IDUA-PGK-BSD Lentivector

**Figure 1:**
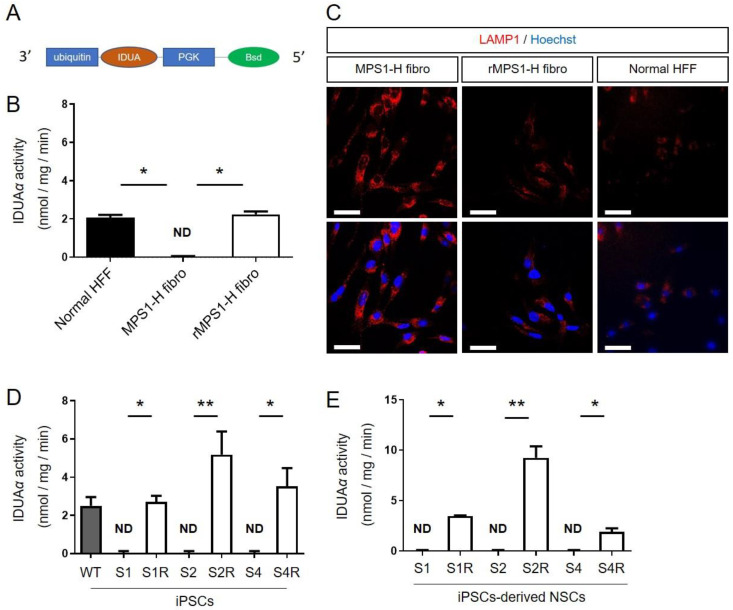
Validation of IDUA rescuing lentivector. (**A**) Schematic representation of the lentiviral vector. (**B**) Iduronidase activity (IDUAα) and (**C**) LAMP1 expression in MPS1-H fibroblasts, MPS1-H rescued fibroblasts and normal human forskin fibroblasts (HFF). (**D**) Iduronidase activity (IDUAα) in the three MPS1-H (S1–S4) and rMPS1-H iPSCs and (**E**) iPSCs-derived NSCs clones (S1R–S4R). Data are shown as mean ± SEM and were analyzed by independent samples *t*-test. * *p* < 0.05; ** *p* < 0.01.

The ability of the lentivector to rescue α-l-iduronidase enzyme (IDUAα) was first validated on MPS1-H patient’s fibroblasts which were used to generate the MPS1-H iPSCs line described in our previous work by Lito et al., [30]. The UBI-IDUA-PGK-BSD lentivector allowed a constitutive expression of α-L-iduronidase enzyme (IDUAα) since *IDUA* is upon ubiquitin (UBI) promotor. An additional transcription unit encoding for blasticidin resistance gene upon human PGK promoter allowed the selection of transduced fibroblasts (Figure 1A).

#### 3.1.1. IDUAα Activity of rMPS1 Fibroblasts

The rescue MPS1-H (rMPS1-H) fibroblasts was first validated by measuring the activity of α-L-iduronidase enzyme (IDUAα). While MPS1-H fibroblasts exhibited no activity of IDUAα (0.01 ± 0.02 nmol/mg/min), fluorimetric assay revealed an activity of IDUAα (2.24 ± 0.07 nmol/mg/min) similar to the one observed in control fibroblasts (2.05 ± 0.08 nmol/mg/min) (Figure 1B). Secondly, we checked the functional impact of IDUAα activity on lysosome storage defects, and more particularly on lysosome-associated membrane protein 1 (LAMP1) accumulation. While LAMP1 immunostaining revealed swollen lysosomes in MPS1-H fibroblasts, lysosomes of rMPS1-H fibroblasts were less affected and are more closely related to normal foreskin fibroblasts (Figure 1C).

#### 3.1.2. IDUAα Activity of rMPS1-H iPSCs

As with fibroblasts, IDUAα activity was restored by the transduction of the three clones (S1, S2 and S4) of MPS1-H iPSCs, with the UBI-IDUA-PGK-BSD lentivector. While all MPS1-H iPSC clones were devoid of IDUAα activity, the fluorimetric 4-MU-iduronide assay revealed a significant activity of IDUAα in the three rescue clones: 2.71 ± 0.32; 5.17 ± 1.21; 3.54 ± 0.94 nmol/mg/min for S1R, S2R, S4R, respectively (Figure 1D). The higher IDUAα activity in rescued clones of MPS1-H iPSCs compared to the one determined in an unrelated wild type control iPSC line (2.51 ± 0.05) must result from the constitutive expression of *IDUA*, which is upon UBI promotor.

#### 3.1.3. IDUAα Activity of rMPS1-H iPSCs-Derived NSCs

Based on the late onset of pathological displays, and more particularly to neuropathological defects, due to the progressive accumulation of glycosaminoglycans (GAG) in lysosomes, we first performed the rescue of MPS1-H iPSCs after their differentiation into neural stem/progenitor cells (NSCs), meaning once they acquired an immature neural phenotype. The transduction of MPS1-H iPSC-derived NSCs restored the activity of IDUAα in the three clones: 3.48 ± 0.07; 9.26 ± 1.96; 1.90 ± 0.60 nmol/mg/min for S1R, S2R, S4R respectively (Figure 1E) while IDUAα activity is not detected in MPS1-H iPSCs-derived NSCs.

### 3.2. Characterization of Rescue rMPS1-H iPSCs

**Figure 2:**
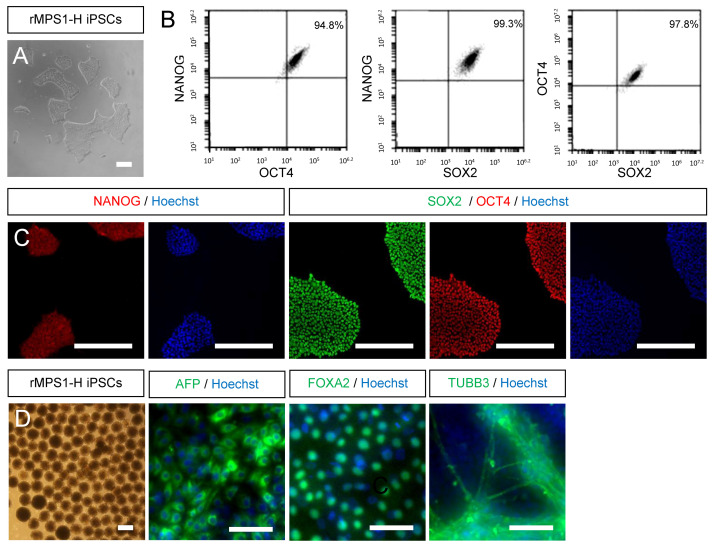
Characterization of rescued MPS1-H iPSC line. (**A**) Rescued MPS1-H iPSC line exhibits morphological aspects of pluripotent stem cells. (**B**) FACS analysis and (**C**) immunofluorescence staining confirm the expression of pluripotent markers NANOG, SOX2 and OCT4. Nuclei was counterstained with Hoechst. (**D**) Validation of the differentiation potential of rMPS1-H iPSC line by immunofluorescence staining of spontaneously differentiated embryoid bodies into the three germ layers: endoderm (AFP), mesoderm (FOXA2), and ectoderm (TUBB3). Nuclei was counterstained with Hoechst. All scale bars = 50 μm.

In order to minimize any variation resulting from different genetic background between iPSCs control lines, we generated an isogenic control line by rescuing the functionality of α-L-iduronidase enzyme (IDUAα) in three clones of MPS1-H iPSCs (S1, S2 and S4). The generated rMPS1-H iPSCs clones (S1R, S2R and S4R), resulting from the blasticidin selection of MPS1-H iPSCs transduced with UBI-IDUA-PGK-BSD lentivector were characterized similarly to MPS1-H iPSCs (S4) [30]. Briefly, rMPS1-H iPSCs presented all specificities of pluripotent stem cells. Cells were tightly packed in colonies and displayed large nucleoli (Figure 2A). FACS analysis (Figure 2B) and immunofluorescence staining (Figure 2C) revealed a high expression of the pluripotency transcription factors OCT4, NANOG and SOX2. Finally, the pluripotency of cells was not affected by the transduction of the lentivector since their spontaneous differentiation, in vitro, in the form of embryoid bodies (EBs) was oriented toward the three germ layers. Indeed, after 24 days, AFP, FOXA2 and TUBB3 immunostaining respectively revealed the presence of endoderm, mesoderm and ectoderm germ layers (Figure 2D).

### 3.3. IDUA Lentiviral Rescue of MPS1-H NSCs Ameliorates Migration In Vitro

**Figure 3:**
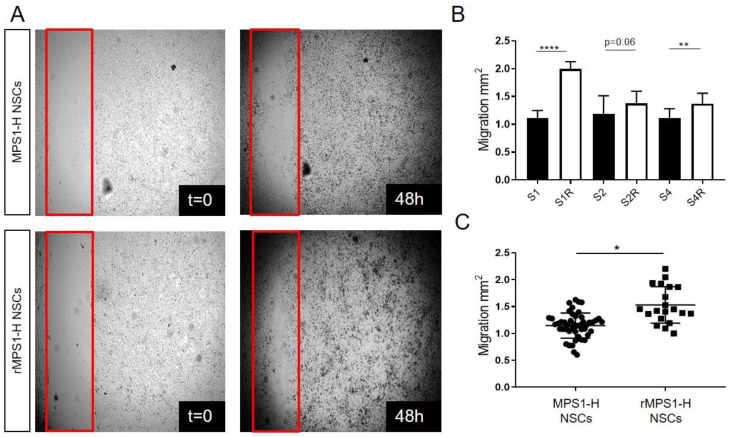
Migration impairment of undifferentiated MPS1-H iPSCs-derived NSCs. (**A**) Acquisitions of MPS1-H and rescued MPS1-H iPSCs-derived NSCs cultures immediately after scratch application (*t* = 0) and 48 h later. The red frame shows scratch boundaries. (**B**) Graphic showing the total migration surface over 48 h of different all clones of MPS1-H and rescued MPS1-H iPSCs-derived NSCs. Data are shown as mean ± SEM and were analyzed by independent samples *t*-test. ** *p* < 0.01; **** *p* < 0.001, *n* = 3. (**C**) Pooled results calculated for MPS1-H iPSCs-derived NSCs vs. rescued ones. Data are shown as mean ± SEM and were analyzed by Welch *t*-test with Moulton correction, *dF* = 3.4; *t* = 5.15. * *p* = 0.01; ** *p* < 0.01; **** *p* < 0.001, *n* = 3.

Neuronal migration occurring during the development of CNS development is a fundamental process leading to severe neurological defects if altered [36]. In order to investigate the functional impact of iduronidase deficiency on the capacity of migration of neural MPS1-H cells, we performed a classic scratch assay using the three clones of MPS1-H iPSC differentiated in NSCs and their corresponding rescued clones (Figure 3A). At 48 h post-scratch, overall covered surface by migrating neural precursors was 1.11 ± 0.13; 1.19 ± 0.32; 1.12 ± 0.16 mm^2^ with S1, S2 and S4 clones respectively versus 1.99 ± 0.13; 1.37 ± 0.22; 1.37 ± 0.19 mm^2^ with the rescued group (Figure 3B). Thus, NSCs displaying a functional activity of IDUAα migrated significantly more (1.53 ± 0.34 mm^2^) than NSCs devoid of IDUAα activity (1.15 ± 0.23 mm^2^) (Figure 3C). This difference in capacity of migration and motility of rescued MPS1-H NSCs compared to MPS1-H NSCs was well illustrated in Video S1.

In parallel, the proliferative capacity of the cells was also assessed during the 3 weeks of NSCs differentiation. As expected, the maturation process was marked by sharp decline in cell proliferation. Indeed, proliferative Ki67 positive cells declined over time from 80% in immature NSCs to less than 15% in differentiated NSCs for both MPS1-H iPSCs-derived NSCs (84.6 ± 6.3% to 10.2 ± 3.9%) and their rescued counterparts (77.6 ± 3% to 11 ± 2.7%) (Data not shown). Thus, cell proliferation is not affected by the mutation of *IDUA*.

### 3.4. Alteration of Neurite Outgrowth during In Vitro MPS1-H Neuronal Differentiation

**Figure 4:**
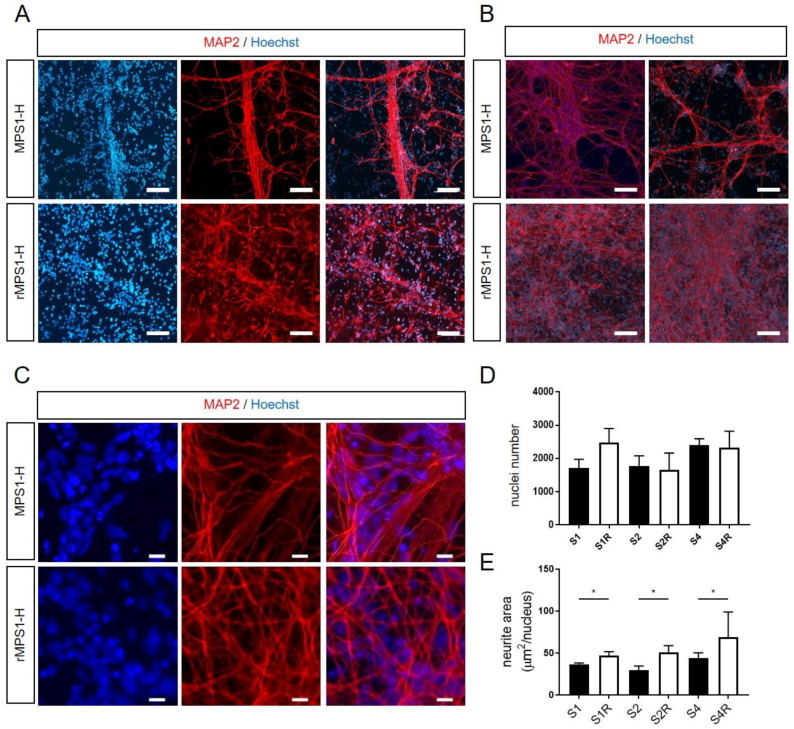
Analyze of neurite outgrowth of MPS1-H iPSCs-derived NSCs and rescued counterparts after 3 weeks of differentiation. (**A**,**B**) Defective neurite outgrowth of MPS1-H iPSCs-derived NSCs compared to rescued NSCs is revealed by MAP2 staining at low magnification (scale bars = 50 µm) and (**C**) high magnification (scale bars = 15 µm) after 3 weeks of differentiation. Nuclei was counterstained with Hoechst. (**D**) Automatic analysis quantified the number of nuclei and (**E**) determined the MAP2-positive neurite surface per nuclei in the different clones. Data are shown as mean ± SEM and were analyzed by independent samples *t*-test. * *p* < 0.01.

Thereafter, we investigated potential pathological effect resulting from IDUAα deficiency during neuronal maturation. Thus, immature rescued and MPS1-H iPSCs-derived NSCs were further differentiated toward neuronal lineage during three more weeks in monolayer, by removing the proliferative growth factors FGF2 and EGF, involved in NSCs stemness. During the 3 weeks differentiation process, we observed a striking difference in the neurite outgrowth of MPS1-H affected neurons compared to rescued neurons. Unlike classic neuronal differentiations or rMPS1-H neuronal cultures, neurites of MPS1-H neurons, stained by the Microtubule-associated protein 2 (MAP2), tended to group together to form bundles and did not spread their neurites over all the culture dish (Figure 4A–C). MPS1-H NSCs cultures showed lesser MAP2 stained area compared to rescued conditions. In order to assess this difference in distribution of neurons, we first counted the number of nuclei (Figure 4D) and normalized the MAP2 positive area to this number (Figure 4E). Thus, an automatic analysis determined the neurite surface per nuclei of each MPS1-H clones: S1: 36.82 ± 1.36 µm^2^/nucleus; S2: 30.64 ± 5.2 µm^2^/nucleus; S4: 46.34 ± 4.1 µm^2^/nucleus; and rMPS1-H clones: S1R: 46.8 ± 4.93 µm^2^/nucleus; S2R: 50.69 ± 8.08 µm^2^/nucleus; S4R: 68.78 ± 30.2 µm^2^/nucleus.

No major variation of nuclei number was observed at the end of NSCs differentiation. This confirmed that MPS1-H and rMPS1-H cells have a similar proliferation rate during neuronal differentiation.

### 3.5. Transcriptome Analysis of MPS1-H Affected and Rescued iPSCs-Derived Neurospheres

**Figure 5:**
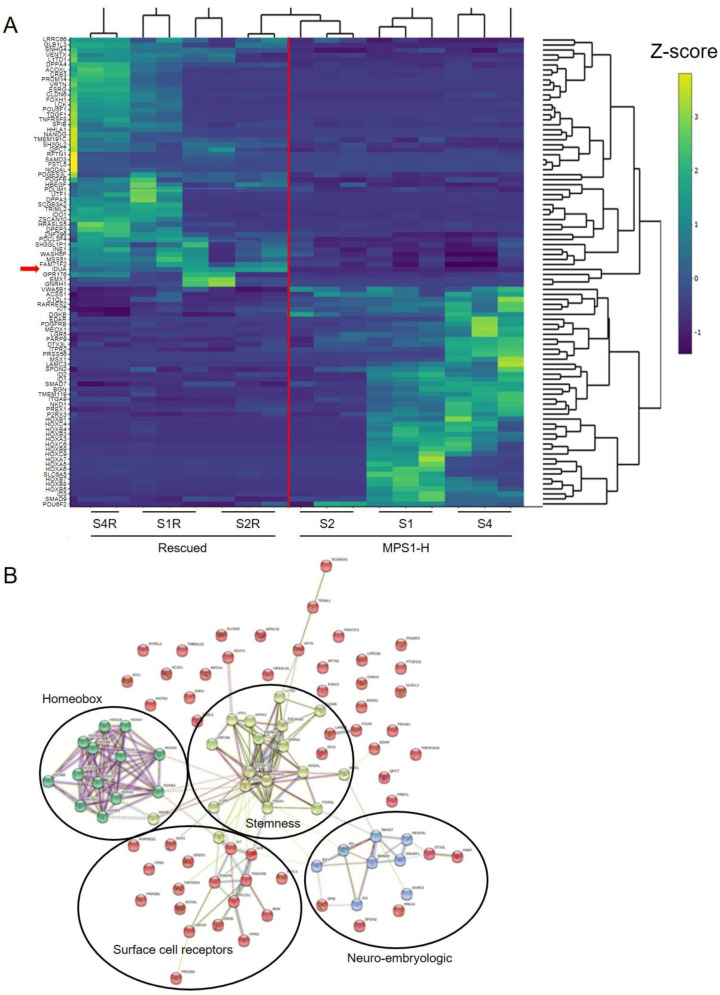
RNA-seq analyzes of 3-week-old MPS1-H and rMPS1-H neurospheres. (**A**) Heatmap displayed of differential expressed genes in MPS I rescued (*left*) and MPS1-H affected clones. Gene expression is represented through Z-score values and according to the brightness of the color code. The red line separates the rescued MPS1-H cluster (*left-half*) from the diseased MPS1-H cluster (*right-half*). The *red arrow* marks the *IDUA* expression. (**B**) Network analysis of differentially expressed genes highlighting four clusters of interacting protein. Each node represents a proteins and edges represent interactions between proteins. Turquoise colored lines represent interactions coming from curated databases; magenta colored lines represent known interactions that have been experimentally defined; other colors are representing predicted interactions, such as gene co-occurrence (blue), gene fusions (red), gene neighborhood (green). The homeobox cluster, stemness cluster, surface cell receptors cluster, neuro embryologic cluster are highlighted. This plot was generated with the string database v.11.0 (http://string-db.org).

In order to identify signaling pathways that may explain some of the neurological dysfunctions observed in MPS1-H, we performed a transcriptomic analysis with RNA-seq on 3 weeks-old neurospheres derived from both rescued and affected MPS1-H iPSCs. The number of genes expressed was 14′839. We found 173 downregulated and 167 upregulated genes in the MPS1-H neurospheres compared to the rescued ones. These genes represented 23 biological processes from KEGG. Key differences were objectivized in association with TGF beta pathway (six genes; *p* = 1.38 × 10^−6^), focal adhesion pathway (four genes; *p* = 0.0106), PI3K-akt signaling pathways (five genes, *p* = 0.0172), Hippo signaling pathway (four genes; *p* = 0.0043), RAP1 pathway (four genes *p* = 0.0127), extracellular matrix (ECM) interaction pathway (two genes; *p* = 0.049), calcium signaling pathway (three genes; *p* = 0.044) (Figure 5A). The 20 most down-regulated and the 20 most upregulated genes are shown in Table 1.

To further highlight connections between the differentially expressed genes, we performed string analysis. This analysis allowed the identification of four main interaction clusters: the homeobox cluster, the stemness cluster, the cell surface receptors cluster (made up mainly of receptor-tyrosine kinases) and the neuro-embryologic cluster (Figure 5B).

## 4. Discussion

Disease models are crucial tools to identify and appreciate pathological mechanisms responsible to the development of a disease [37]. Therefore, it is essential to have a relevant study model which must reproduce the pathological features of the disease to be able to design novel therapeutic strategies. Despite the possibility to obtain primary cells from patients affected from various inherited diseases, in vitro disease modeling was highly limited during the past due to the difficulties to maintaining these cells in culture. Cardiovascular or neurological diseases modelling was even more challenging due to the additional limitation linked to the access to patient’s tissues. The discovery and the isolation of human embryonic stem cells (hESCs) [38] in 1998, first allowed to lift a part of these constraints. Indeed, their ability to undergo unlimited self-renewal in culture followed by their differentiation into multiple cell types of the body [39,40,41,42] allowed the establishment of models for many diseases, including muscular or neurological diseases [43,44,45,46,47]. However, the possibility to model any disorder, including the one not tested during preimplantation genetic diagnosis (PGD), such as neurodegenerative diseases [48,49,50,51,52] was made possible by the recent discovery of iPSCs by Yamanaka [53], allowing the conversion of somatic cells into pluripotent stem cells [54,55].

In this study, we took advantage of the capacity of differentiation of an iPSCs line, previously derived from a MPS1-H child [30], to investigate the effect of IDUAα deficiency during neural development which could explained some neural disorders observed on MPS1-H patients. Since RNA sequencing was one of the mainstays of our strategy, we needed a control iPSCs line presenting the most similar genetic background to our MPS1-H iPSC line to identify gene variations resulting from the disease-associated mutation rather than genetic background noise [31,56]. As we did not dispose to an iPSCs control line from a parent or a close relative, we generated an isogenic line by rescuing *IDUA* expression in our MPS1-H iPSCs line by a lentivector [32]. Although recent advances in genome editing technology would have corrected the mutation without insertion of new DNA sequence, or even producing an isogenic MPS1-H line by introducing a specific mutation into one of our control iPSCs line [57,58], this technology remains challenging for the non-expert operators. Nevertheless, similarly to the work of Tolar et al., [59] the self-renewal and the differentiation potential of our isogenic control iPSCs clones was not altered by the integration of the lentivector. Moreover, our results reported a similar level of IDUAα activity in rescued cells compared to control ones (Figure 1).

Differentiation of MPS1-H affected iPSCs clones into neuronal cells revealed both migration defects and alteration of neurite outgrowth compared to their three rescued counterparts. Our results revealed a similar cell proliferation between MPS1-H iPSCs and rMPS1-H iPSCs during neuronal differentiation, the accumulation of GAGs, resulting for IDUAα deficiency, remains the most evident hypothesis that could explain these defects as it was highlighted in MPS3 also known as Sanfilippo syndrome [18,19,20]. Indeed, CS and HS proteoglycans which are the main components of the cell surface and ECM in the brain [60], are known for their binding properties and are consequently involved in multiple biological pathways [61], including cell migration, cell adhesion and cell polarization processes [62,63]. By binding axon guidance molecules such as ephrin, netrins, semaphorins and Slit families, they must highly disrupt cell migration and neurite outgrowth [64] during brain development. Moreover, many studies have highlighted the crucial role of ECM in neurodevelopment and have even linked the appearance of neurodegenerative disorders to its disruption [65].

A striking result of our study is the magnitude and the coherence of the changes in gene expression. Basically, changes in gene expression concerned 23 biological processes and were identified as four clusters by string analysis. These changes make sense in the context of the cellular alterations observed in our study, as well as the neuropathological features described in MPS1-H patients. Indeed, focal adhesion pathway, RAP1 pathway [66] and ECM interaction pathway point towards cell motility and the migration defect identified above since the alteration in expression of the Hippo pathway [67] and homeobox genes and neuro-embryologic genes point toward the neuroanatomical defects.

Taken together our results demonstrate a defect in migration of neural precursor cells, as well as massive changes of gene expression in neuronal cells derived from MPS1-H iPSCs. The relationship between migration defect and changes in gene expression are most likely bidirectional: altered adhesion and ECM gene expression is likely to precede an alteration of cell migration, but impairment of cell migration can also lead to altered gene expression. Further studies will be necessary to define the contribution of these individual factors to MPS1-H neurological disease. We are confident that our study provides a strong framework to advance for such an endeavor.

## Figures and Tables

**Table 1:** RNA-seq of MPS1-H iPSCs derived neurospheres normalized on rescued neurospheres.

Upregulated Genes			Downregulated Genes		
Gene Name	Log FC	False Discovery Rate (FDR)	Gene Name	Log FC	False Discovery Rate (FDR)
*HOXA3*	8.498	0.014	*LINC01405*	−5.762	0.038
*HOXA7*	7.852	0.032	*ZFP42*	−5.879	0.061
*HOXB9*	7.789	0.030	*ACOXL*	−6.305	0.039
*HOXA5*	7.530	0.032	*JAKMIP2-AS1*	−6.335	0.034
*EDAR*	7.514	0.036	*ZSCAN10*	−6.423	0.034
*SOST*	7.251	0.058	*PRDM14*	−6.602	0.034
*HOXC9*	7.192	0.030	*LINC00428*	−6.659	0.034
*HOXC6*	6.280	0.039	*VRTN*	−6.694	0.039
*HOXA6*	6.054	0.046	*DPPA3*	−6.734	0.032
*GDF7*	5.721	0.079	*FOXH1*	−6.858	0.032
*HOXB3*	5.710	0.046	*LINC00678*	−7.005	0.032
*SLC6A5*	5.664	0.042	*NODAL*	−7.012	0.039
*HOXB5*	5.618	0.034	*TDGF1*	−7.073	0.036
*PRSS56*	5.535	0.039	*HHLA1*	−7.299	0.027
*HOXB4*	5.501	0.046	*LINC01108*	−7.349	0.030
*HOXB8*	5.455	0.046	*TRIML2*	−7.479	0.030
*HOXB7*	5.296	0.036	*SCGB3A2*	−7.745	0.030
*HOXC4*	5.226	0.039	*LOC101929194*	−7.835	0.078
*ATOH1*	5.170	0.085	*POU5F1*	−7.861	0.032
*HOXB1*	5.048	0.042	*ESRG*	−8.122	0.032

Values in the table refer to the comparison of neurospheres derived from diseased vs. rescued MPS1-H iPSCs. The term “upregulated genes” refers to an expression that is higher in diseased vs. rescued neutrospheres.

## Supplementary Materials

The following are available online at https://www.mdpi.com/2073-4409/9/12/2593/s1, Video S1: Time laps of scratch test performed on undifferentiated MPS1-H iPSC-derived NSCs.
