# Supplementary figures and images for: Induced Pluripotent Stem Cells to Understand Mucopolysaccharidosis. I: Demonstration of a Migration Defect in Neural Precursors
Source: Cells. 2020 Dec 3;9(12):2593. doi: 10.3390/cells9122593 (PMC7761689; doi:10.3390/cells9122593)

## Slide 1
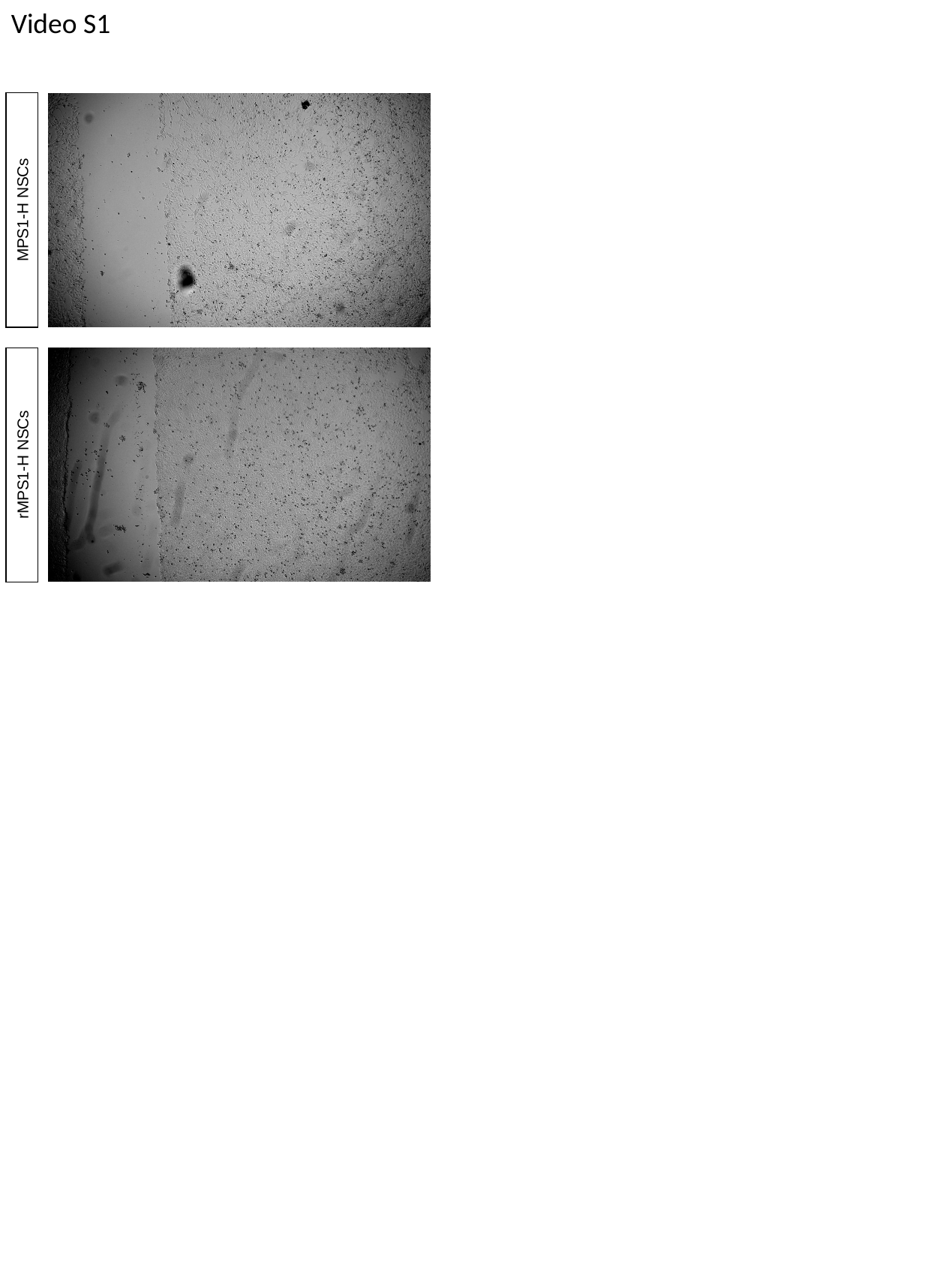

Video S1
MPS1-H NSCs
rMPS1-H NSCs

Supplement: Supplementary file 1 [file cells-09-02593-s001.zip › Supplementary Video S1.pptx]
